# Shadows in the forest: Uncovering unusual colouration records in mammals from the Ecuadorian Tropical Andes

**DOI:** 10.3897/BDJ.12.e137463

**Published:** 2024-11-20

**Authors:** Elias Viteri-Basso, Juan Pablo Reyes Puig, Carolina Reyes-Puig, Gorky Ríos-Alvear

**Affiliations:** 1 Universidad San Francisco de Quito USFQ, Colegio de Ciencias Biológicas y Ambientales, Instituto de Biodiversidad Tropical IBIOTROP, Laboratorio de Zoología Terrestre, Quito 170901, Ecuador Universidad San Francisco de Quito USFQ, Colegio de Ciencias Biológicas y Ambientales, Instituto de Biodiversidad Tropical IBIOTROP, Laboratorio de Zoología Terrestre Quito 170901 Ecuador; 2 Ecominga Foundation, Baños, Ecuador Ecominga Foundation Baños Ecuador; 3 Instituto Nacional de Biodiversidad, Quito, Ecuador Instituto Nacional de Biodiversidad Quito Ecuador; 4 Fundación Oscar Efrén Reyes, Baños, Ecuador Fundación Oscar Efrén Reyes Baños Ecuador; 5 Universidad San Francisco de Quito, Quito, Ecuador Universidad San Francisco de Quito Quito Ecuador; 6 CIBIO Centro de Investigación em Biodiversidade e Recursos Genéticos, Porto, Portugal CIBIO Centro de Investigación em Biodiversidade e Recursos Genéticos Porto Portugal; 7 Grupo de Biogeografía y Ecología Espacial (BioGeoE2). Universidad Regional Amazónica Ikiam, Tena, Ecuador Grupo de Biogeografía y Ecología Espacial (BioGeoE2). Universidad Regional Amazónica Ikiam Tena Ecuador

**Keywords:** colour anomalies, colouration, melanism, *Leopardus* spp., activity patterns, mammals, camera trap, CELS

## Abstract

Variations in colouration patterns have been reported in numerous wildlife species, particularly birds. However, the increased use of camera traps for wildlife monitoring has enabled the detection of elusive species and phenotypic variations that might otherwise go undetected. Here, we compiled records of unusual colouration patterns in terrestrial mammals, documented through camera-trap studies over a 12-year period in the Llanganates-Sangay Connectivity Corridor, in the Tropical Andes of Ecuador. We identified colour variations in seven species of terrestrial mammals, including disorders, such as melanism, white spotting/ piebaldism, xanthocromism and progressive greying. Notably, we reported a high prevalence of melanism in wild populations of the clouded oncilla, along with observations on the species' activity patterns. Approximately half of the recorded clouded oncillas were melanistic. We detected significant differences in activty patterns between melanistic and non-melanistc clouded oncilla, with melanistic morphs showing a peak of activity between 3 a.m. and before dawn. The proportion of melanistic individuals suggests that melanism is widespread throughout the corridor. However, its impact on the species' fitness remains unclear.

## Introduction

Camera trapping is an effective and non-invasive technique for wildlife monitoring. It provides insights into the species behaviour, spatial distribution occurrence, predation and other ecological features which are difficult to observe directly (Delisle et al. 2021, Macas-Pogo et al. 2023). Camera trapping allows us to collect information on elusive species, which commonly occur at low densities and whose museum records are scarce, like medium and large-size mammals (Tobler et al. 2008). In addition, it allows for the identification of less frequently occurring phenotypic traits that may not be detected using different sampling methods. One of these observable traits is the presence of unusual colouration patterns, which can arise from variations in diet or the expression of specific mutations often found in genetically isolated populations (Hubbard et al. 2010).

Colour variation can be found in reptiles, birds, mammals, fish and even anurans. It is typically characterised by differences in the concentration of melanin and, although less common in mammals, by variations in the absorption of carotenoid pigments from their diet (van Grouw 2013, Lindgren et al. 2015, Galván et al. 2016). Carotenoid levels are affected by diet, so, when resolving the dietary deficiencies, unusual colouring patterns disappear (van Grouw 2006). On the other hand, melanin is a family of biopolymers distributed throughout epidermal tissue. Its synthesis relies on the amino acid tyrosine and has a genetic basis. It can be inhibited by mutations that affect the enzyme tyrosinase (van Grouw 2006, Fertl and Rosel 2009, Lindgren et al. 2015). Melanin is responsible for dark colour tones, which can vary depending on the pathways mediated by the presence of thiol compounds. In the absence of thiols, eumelanin is produced, while the presence of cysteine results in pheomelanin synthesis (Prota 1992). Eumelanin pigments result in black, grey and brown tones, while yellow to reddish pigments are due to pheomelanin (Ito and Wakamatsu 2003, van Grouw 2006, van Grouw 2013). However, the colour expression depends on the combination of both melanin pigments (Prota 1992, Ito and Wakamatsu 2003). The lack or defect in tyrosinase prevents melanin synthesis, thereby causing albinism. As a result, the albino's body appears colourless, while the skin and eyes exhibit a reddish hue due to the visibility of internal blood vessels. Conversely, melanistic phenotypes may arise from disorders in melanin deposition, resulting from either variation in the quantity or type of melanin produced (i.e. eumelanin or pheomelanin) often leading to darker colouration in melanistic individuals (Caro 2005, van Grouw 2006, van Grouw 2021). When melanin is normally produced, but does not deposit in cells, it results in white colouration due to the lack of pigment on the body, while the eyes and skin maintain normal colouring patterns (i.e. leucism) (van Grouw 2006, Lucati and López-Baucells 2017, van Grouw 2021). Similarly, white spotting is the congenital absence of functional melanocytes due to various mutations, including the piebald gene, which has led to generically call this disorder piebaldism. White spotting is characterised by distinct white marks resulting from the localised absence of melanocytes in the hair follicles or epidermis (Fertl and Rosel 2009, Lamoreux et al. 2010, Abreu et al. 2013, Lucati and López-Baucells 2017, Streeting et al. 2023). From now on, we will refer to this condition explicitly as piebaldism to avoid confusion with other studies (Lucati and López-Baucells 2017). Progressive greying, often confused with partial leucism, refers to the gradual loss of pigmentation that occurs with successive moults. It results from a failure to incorporate melanoblasts into new hair growth, leading to a lack of pigmentation as individuals age. Consequently, affected individuals display non-pigmented diffuse areas throughout the body (Lamoreux et al. 2010).

Gloger’s rule states that mammals in the Tropics are more likely to exhibit dark colourations and, thus, are prone to develop melanism (Caro 2005, Caro and Mallarino 2020, Mooring et al. 2020), with factors like temperature, humidity, forest cover and vegetation density being the more influential variables (Delhey 2019, Caro and Mallarino 2020, Mooring et al. 2020). Unusual colouration patterns in mammals can be detrimental to their fitness (i.e. survival rate, reproductive success and energy contribution to the ecosystem) (Peacock 2011), by making individuals more visible to predators, sometimes less sexually desirable, presenting higher heat absorption levels, as well as increasing the predator's exposure to their prey, impacting on their survival probability (van Grouw 2006, Abreu et al. 2013, Mooring et al. 2020, Streeting et al. 2023). Moreover, some genetic mutations that cause unusual colouration patterns are pleiotropic; they affect multiple developmental processes beyond colour, which may indicate underlying physiological alterations (Lamoreux et al. 2010).

Reporting unusual colouration in wildlife enhances our understanding of various aspects of their behaviour and biology. This includes describing the colour patterns of different species, clarifying species taxonomy, studying how colour affects survival rates and sexual selection and exploring its influence on predator-prey interactions, predatory success and the physiological implications of colour variation at both individual and population levels (van Grouw 2006, Abreu et al. 2013, Caro and Mallarino 2020, Aximoff et al. 2021). For instance, it is crucial to identify whether certain colour variations are more prevalent in populations exposed to specific factors and to determine their impact on species vulnerability.

The Tropical Andes are considered a biodiversity hotspot (Myers et al. 2000). In Ecuador, the eastern slopes of the Andes are deemed a priority area for conservation due to their rugged topography and well preserved natural habitat remnants which support high levels of biodiversity and endemism (Jost 2004, Reyes-Puig et al. 2022). We report records of unusual colouration in medium-sized mammals from the Llanganates-Sangay Connectivity Corridor (CELS) in Ecuador. We gathered independent camera-trap records of distinct colouration anomalies in various mammal species from different studies within CELS and compared the proportion of these anomalies in each species. We also assessed the effect of these anomalies on the activity patterns of felids.

## Materials and methods

### Study area

We conducted the study in the eastern slopes of the Tropical Andes in Ecuador, in the Llanganates-Sangay Connectivity Corridor (CELS) (Ríos-Alvear and Reyes-Puig 2015, Ríos-Alvear et al. 2024). The CELS encompasses 92,148 hectares in the transition zone between the Ecuadorian Amazon and the Andes, ranging from 760 to 3,812 elevation m. The strategic location of CELS promotes the habitat connectivity between the Llanganates and Sangay National Parks across a human dominated landscape. It comprises areas of natural vegetation in 90% of its total extension which have allowed the establishment of private conservation initiatives in half of its extension. Other land uses include agriculture and cattle ranching areas (Ríos-Alvear et al. 2024). The rugged topography of CELS shapes the formation of biogeographic barriers, favouring species endemism (Jost 2004, Haynie et al. 2006, Reyes-Puig et al. 2022) and contributes to the occurrence of natural habitat remnants that serve as critical habitats for threatened species (Ríos-Alvear et al. 2024). The CELS is characterised by hyper-humid weather conditions, with a rainfall regime that exceeds 5000 mm annually (Ilbay-Yupa et al. 2021) and mean temperature of 19°C.

### Camera-trap records

We compiled camera-trap records from four studies carried out between 2011 and 2024 in CELS, as well as casual records shared by park rangers from the Sangay National Park, one museum record and one record of a melanistic deceased *L.pardinoides* that was run over. Studies were conducted with specific objectives, varying sampling efforts, spatial coverage and at different elevation ranges (Table 1). Nevertheless, camera-trap placement was along wildlife trails within natural vegetation. All studies placed their camera-trap stations in the exact same location during the study period. We consider as an independent record those pictures or videos of the same species taken at the same camera-trap station with an interval of more than 60 minutes (Rovero and Marshall 2009). Cameras were operational 24 hours a day without the use of bait. Pictures of unusual colourations were observed and chosen for taxonomic identification and to validate the colour pattern.

### Identification of unusual colourations

**Figure 1.**
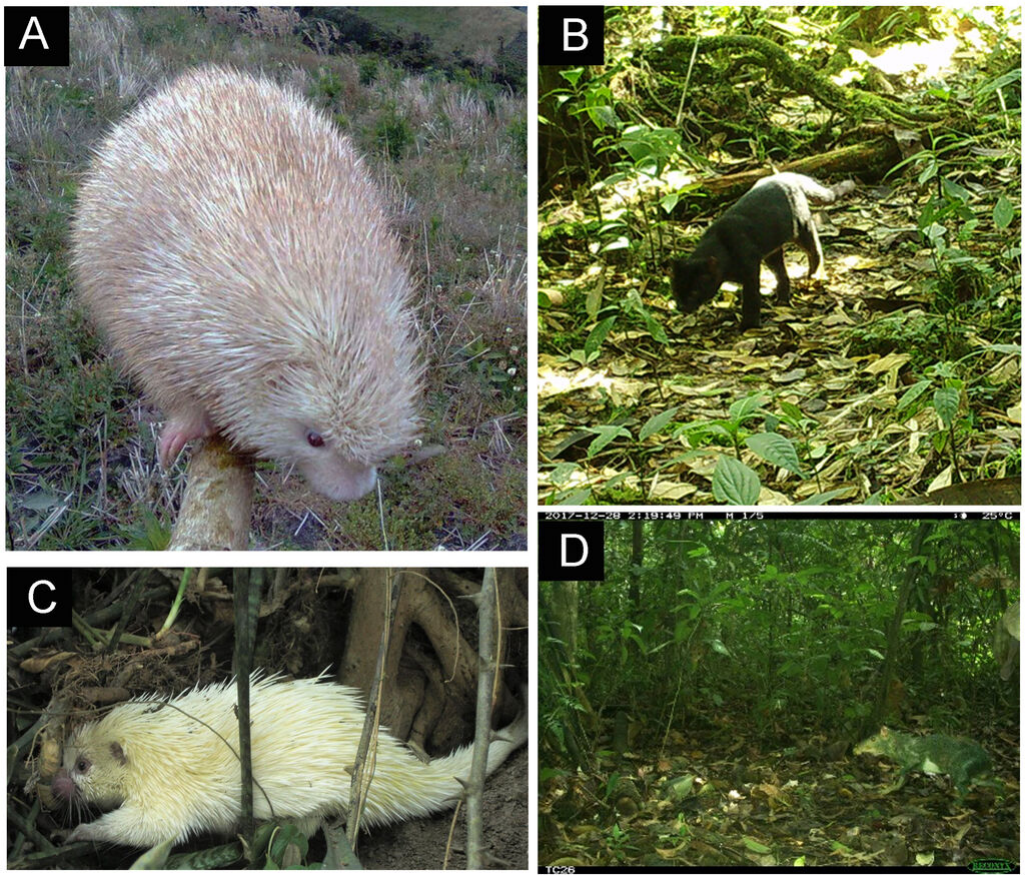
Criteria for identifying unusal colouration following the phenotypic identification of Lucati and López-Baucells (2017). **A.** Albino *Coendourufescens* with completely white fur due to the melanin absence along with pinkish or reddish eyes and skin (Picture from Romero et al. (2018)). **B.** Melanistic clouded oncilla exhibiting dark colouration due to excessive melanin. Melanism can occur throughout the entire body or in specific areas (i.e. partial melanism) (Picture from this study). **C.** Leucistic *Coendouprehensilis* exhibiting pale to white colouration throughout its body, while retaining normal pigmentation in its eyes and skin (Picture from Romero-Briceño and González-Carcacía (2020)). **D.** *Dasyproctafuliginosa* specimen showing white spotting, a condition commonly known as piebaldism. The specimen displays a clearly defined and localised absence of pigmentation ventrally, resulting in a distinct white patch (Picture from Mejía Valenzuela (2019)).

Due to the fact that most of our records were obtained from camera traps, which are subject to variations in image quality and resolution, we categorised the unusual colouration into five main categories. Four of them were phenotypically identified following Lucati and López-Baucells (2017) (Fig. 1) and the fifth category corresponds to progressive greying (Lamoreux et al. 2010). We assumed that leucism and piebaldism differ due to varying degrees of pigmentation absence, with piebaldism exhibiting well-defined patches lacking pigmentation, while progressive greying appears as diffuse depigmentation along the entire body. During the identification process, each author reviewed the records individually and then discussed their findings to agree upon the identifications. When we could not reach a consensus on the species' taxonomic identification (particularly in felids) or the identification of its corresponding unusual colouration pattern, we resolved the discrepancies by consulting experienced colleagues who acted as external reviewers.

### Activity patterns

Activity patterns have been defined as “the temporal structure of physical activity and sedentary behaviour [movement behaviours] accumulated over a specified time period during the waking hours” (Ridgers et al. 2023). However, two related, but distinct definitions are involved: activity patterns and levels. Activity patterns correspond to the distribution of an animal's activity based on light and environmental factors variations throughout the day, with three general categories: diurnal, nocturnal and crepuscular (Ashby 1972). Activity levels represent the proportion of time during which the organism is considered active (Rowcliffe et al. 2014). We agreed that an animal was considered "active" if it was recorded as "moving" in front of the camera trap (e.g. walking, running, foraging or hunting).

We analysed the activity patterns of species with the most independent records showing unusual colouration, specifically the clouded oncilla (*Leoparduspardinoides*) and margay (*L.wiedii*), which co-occur in the study area. We extracted the total independent records of non-melanistic individuals and those exhibiting melanistic colouration. The activity patterns of the non-melanistic and melanistic independent records were analysed with the package 'Activity' (Rowcliffe 2023) in R version 4.3.3 (R Core Team 2023). We fitted kernel density functions to wildlife activity time data to estimate activity levels and patterns from the observed independent records. We converted the dates and times into radians, then calculated the circular mean for each dataset (total records of clouded oncilla, common coloured clouded oncilla, melanistic clouded oncilla and margay total/common colouration). This allowed us to represent the average direction of the radian values for each group. To estimate the species activity, we used the 'fitact' function with 9,999 repetitions and, for comparing the activity levels between datasets, we performed the Wald test using the 'compareAct' function. Additionally, to compare circular distributions, we used the 'compareCkern' function with 9,999 repetitions, which calculates the overlap index "Dhat4" for pairs of fitted distributions. We estimated the overlap coefficient (Δ) between datasets, considering 0 for no overlap and 1 for total overlap (Ridout and Linkie 2009). We used Δ_4_ following the recommendations of Meredith and Ridout (2017) for small sample sizes. We created plots using the "overlapPlot" function from the "overlap" package (Meredith et al. 2024) to visualise kernel density. For our plots, we set sunset at 6:00 pm and sunrise at 6:00 am, as temporal variations related to sunrise and sunset are minimal in continental Ecuador and have been applied in similar studies (Salvador and Espinosa 2016, Espinosa and Salvador 2017).

## Results

### Unusual colouration records

**Figure 2.**
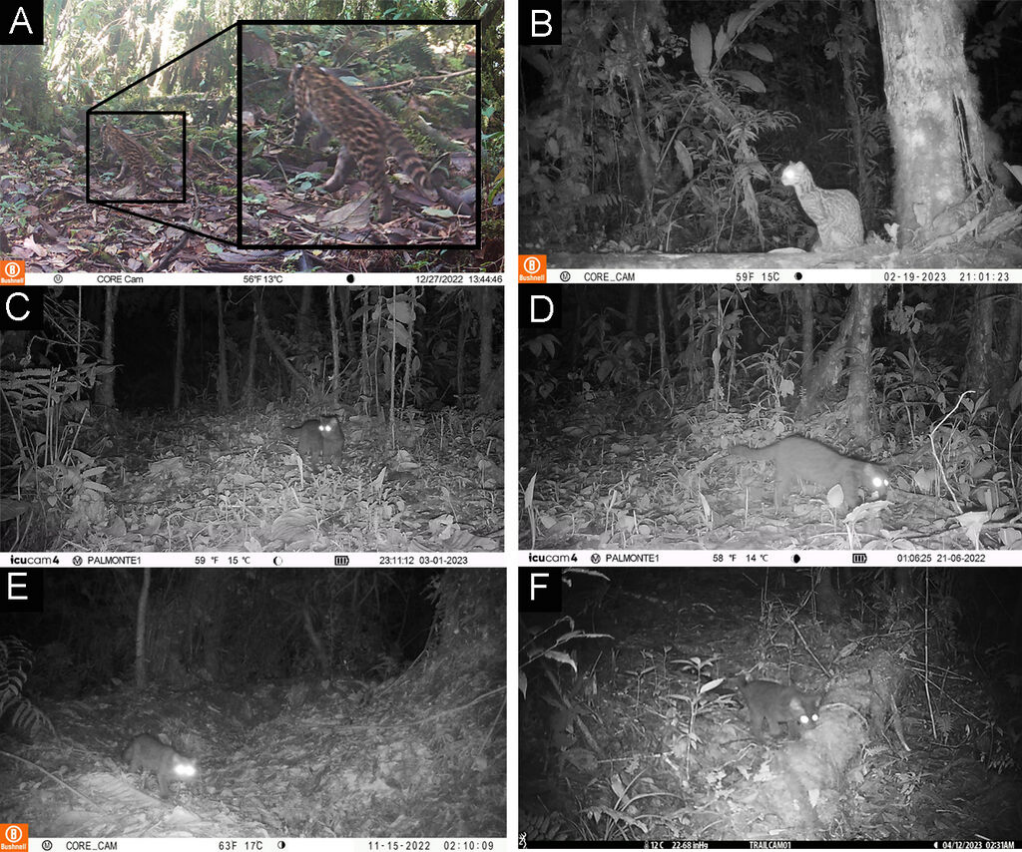
Records of wild felids. **A.** *Leoparduspardinoides* recorded during the daytime. **B.** *Leopardus* sp. recorded at night. **C.** Melanistic margay (*L.wiedii*). **D - F.** Melanistic clouded oncilla (*L.pardinoides*).

**Figure 3.**
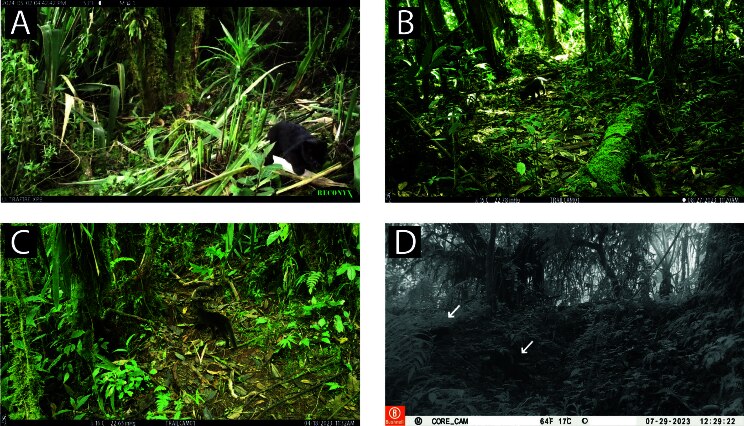
Records of melanistic individuals of clouded oncilla active during the daytime.

**Figure 4.**
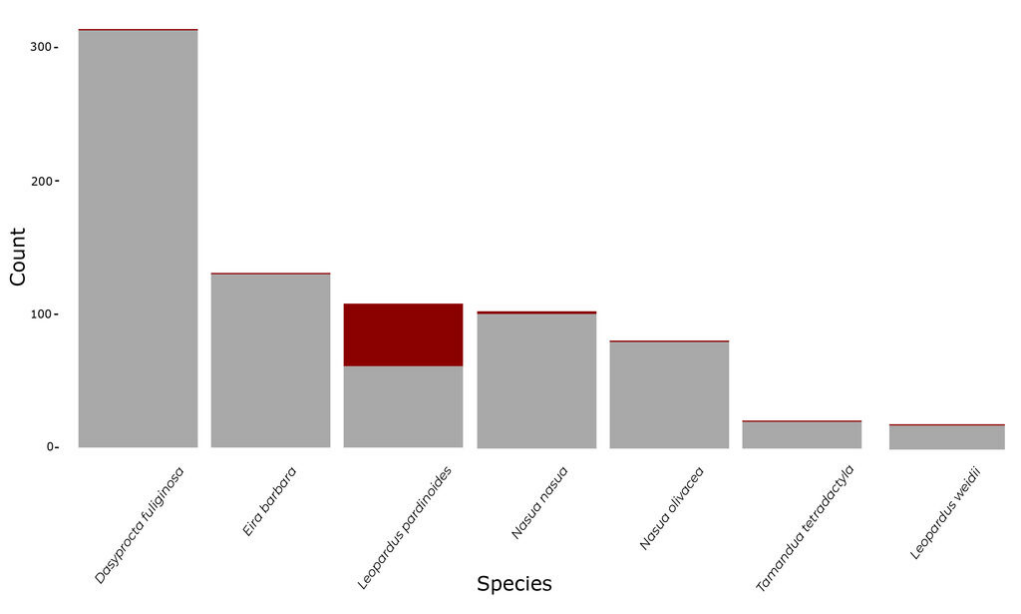
Proportion of independent records of individuals with unusual colouration compared to those with common colouration for each species (based on the count of independent records reported for each species in this article). Red colouration represents the independent camera-trap records with unusual colouration.

We compiled a total of 57 unusual colouration records encompassing seven different species: black agouti (*Dasyproctafuliginosa*), tayra (*Eirabarbara*), clouded oncilla (*Leoparduspardinoides*), margay (*Leoparduswiedii*), brown-nosed coati (*Nasuanasua*), western mountain coati (*Nasuaolivacea*) and southern tamandua (*Tamanduatetradactyla*). Fifty-one of these were records of melanistic felids (*Leopardus* spp.). We documented only one independent record of a melanistic margay (Fig. 2C). The species with the most records was the clouded oncilla, with a total of 49 melanistic records (Fig. 2**D-F**), some of which occurred during the daytime (Fig. 3**A-C**), with one standout observation of two melanistic individuals together (Fig. 3D). The proportion of independent records displaying unusual colouration was low for most of the species (5.2% for margay, 4.7% for southern tamandua, < 2% in the other species), except for the clouded oncilla in which almost half of the total records correspond to melanistic individuals (43.5%) (Fig. 4). We also included a camera trap record of a melanistic clouded oncilla, documented by rangers in Sangay National Park, along with a casual observation of a road-killed melanistic clouded oncilla on the Guamote-Cebadas-Macas road (lat. -2.183529, long. -78.330431).

**Figure 5.**
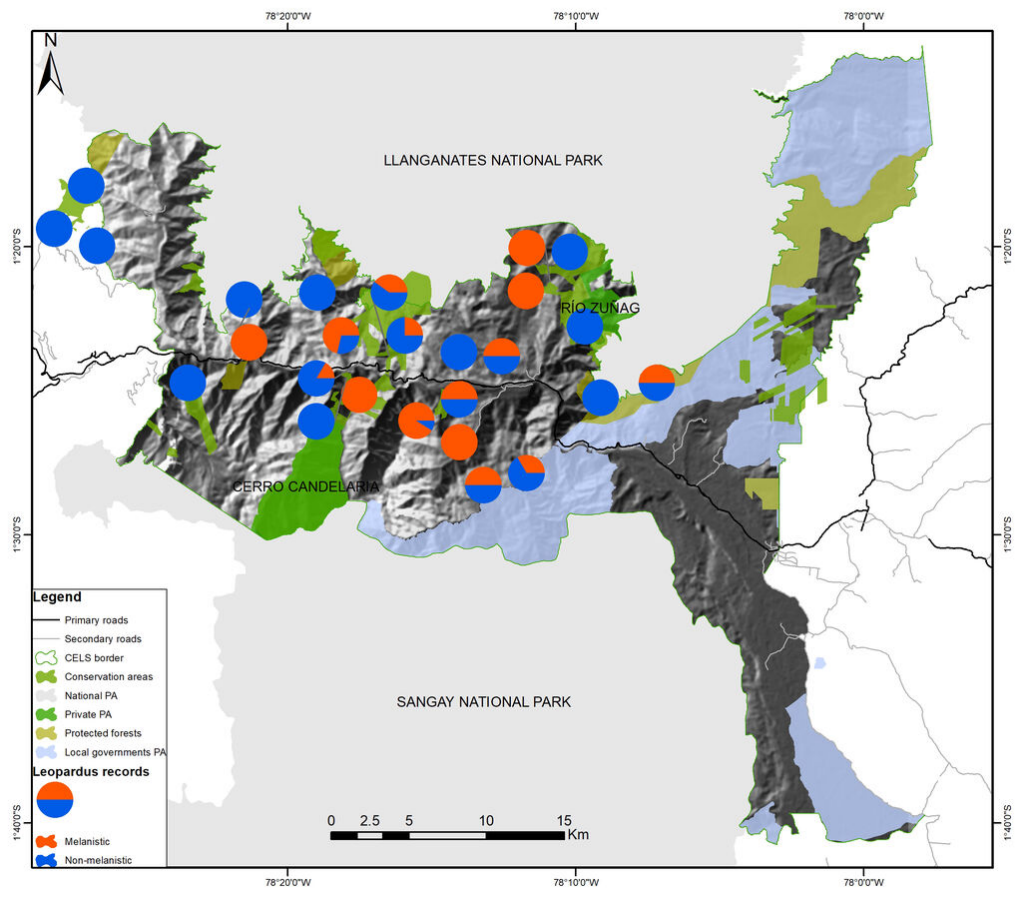
Records of the clouded oncilla (*Leoparduspardinoides*) within CELS.

We recorded both melanistic and non-melanistic clouded oncillas at nearly 30% of our sampling locations within CELS. Notably, melanistic individuals were documented at 44% of the sampling sites across the species' distribution range. Interestingly, in 15% of our sampling locations, only melanistic clouded oncillas were observed (Fig. 5).

**Figure 6.**
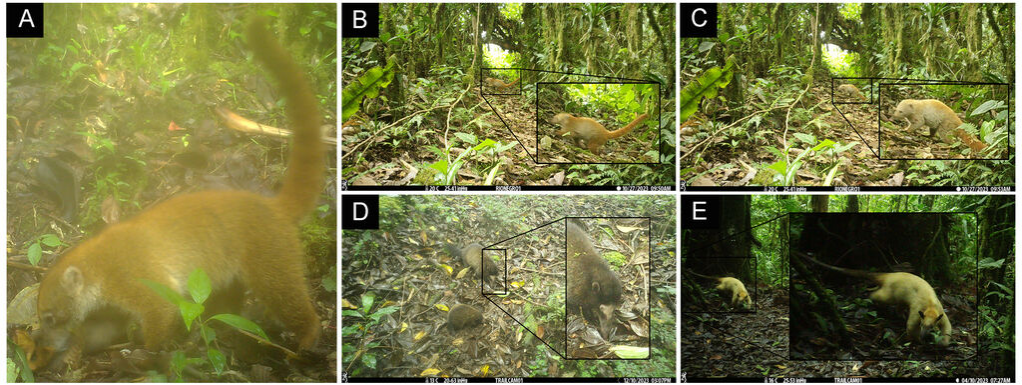
Records of unusual colouration in terrestrial mammal species. **A.** Brown-nosed coati displaying the typical colouration pattern (*Nasuanasua*); **B-C.** Brown-nosed coatis exhibiting progressive greying in the flank, head and part of the legs; **D.** Western mountain coati (*Nasuaolivacea*) exhibiting white spotting/piebaldism on the snout and part of the head, including the right eye; **E.** Xanthochromism in Southern tamandua (*Tamanduatetradactyla*).

**Figure 7.**
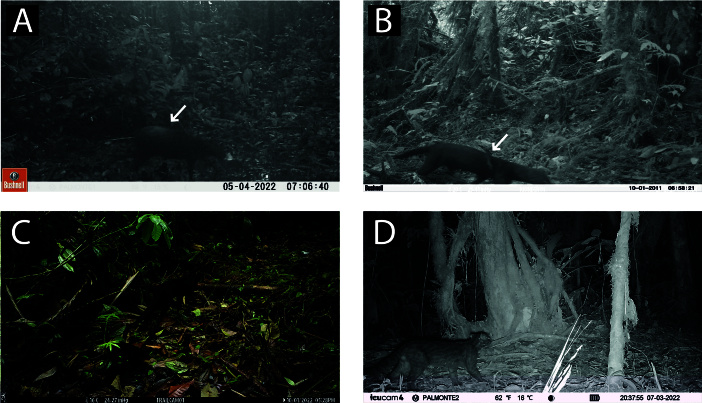
Records of unusual colouration in terrestrial mammal species from CELS. **A.** Black agouti exhibiting white spotting/ piebaldism in the lower back; **B.** Tayra with a white spot/piebaldistic mark in the back of its right shoulder; **C.** Melanistic clouded oncilla active at the daytime; **D.** Melanistic clouded oncilla with visible dark rosettes at the flank.

We documented unusual colouration patterns in several terrestrial mammal species that, to our knowledge, have not been previously reported in the study area (Figs 6, 7). For example, the brown-nosed coati usually has a body colouration ranging from pale yellow to brown tones, with dark brown legs and conspicuous dark rings along the tail (Gompper and Decker 1998) (Fig. 6A). The specimens in Fig. 6 (B and C) display a noticeable lack of pigmentation across the body, significantly differing from the typical colouration patterns reported for these species (Gompper and Decker 1998, Tirira 2007, Hayssen 2011, Brito et al. 2023). We recorded three species with piebaldism, a western mountain coati with a piebaldistic mark on the front face (Fig. 6D), a black agouti individual exhibiting a well-defined mark in the dorsum, and a tayra with an elongated piebaldistic mark on the shoulder (Fig. 7A and B). We recorded a specimen of a southern tamandua lacking the dark vest shape and exhibiting a yellowish colouration in the back of the head and dorsum which is different from the typical colouration (Hayssen 2011) (Fig. 6E). Additionally, we observed variations amongst melanistic individuals, with clouded oncillas displaying different patterns: one exhibiting completely dark colouration (Fig. 7C) and another showing dark colouration with subtle visible rosettes in the background (Fig. 7D).

### Activity patterns

**Figure 8.**
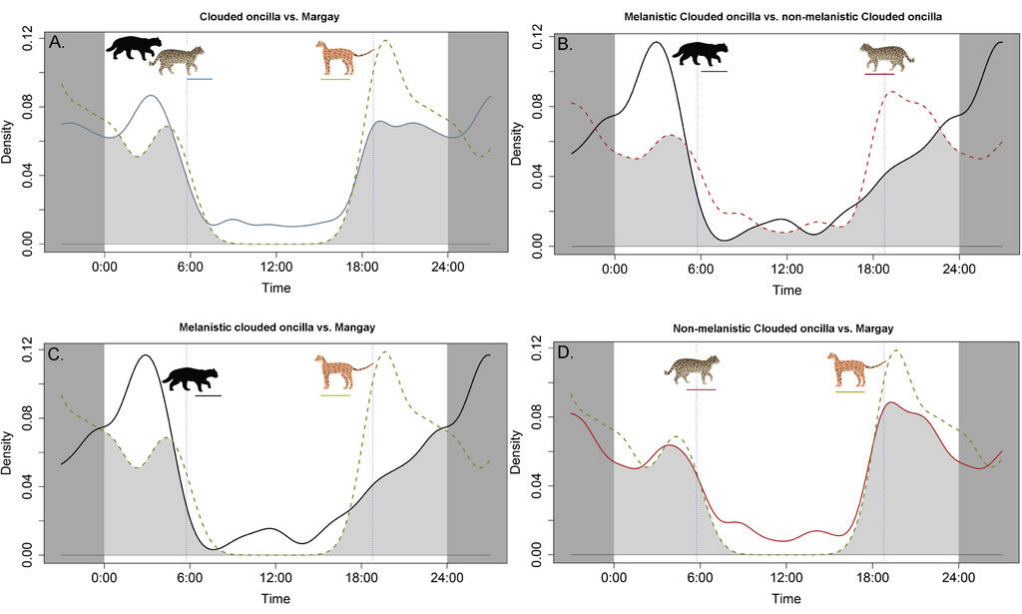
Activity patterns of clouded oncilla and margay from CELS. **A.** Activity patterns of clouded oncilla (blue line) and margay (green dashed line) according to the total of records. Shaded in grey is the overlapping of activity patterns by hours; **B.** Activity patterns of melanistic (black line) and typically colouration records (red dashed line) of clouded oncilla; **C.** Activity patterns of melanistic records of clouded oncilla (black line) and typically colouration records of margay (green dashed line); **D.** Activity patterns of independent records with typically colouration only, clouded oncilla (red line) and margay (green dashed line).

Both *Leopardus* species are primarily nocturnal, although the clouded oncilla shows a slight increase in activity during the day compared to the margay (Fig. 8A). However, when we analysed the activity patterns of the clouded oncilla in more detail, melanistic individuals showed a distinct peak of activity around 3 a.m., where their activity is more concentrated. In contrast, individuals with common colouration are most active between 6:30 and 9 p.m. (Fig. 8B). Similarly, margays also exhibit a peak in activity around 7 p.m. (Fig. 8C and D).

Regarding activity levels between data-sets, we detected no significant differences between the clouded oncilla and margay (W = 1.872, p = 0.171), between melanistic and typically coloured clouded oncilla (W = 1.435, p = 0.230), between melanistic clouded oncilla and margay (W = 0.003, p = 0.954) or between typically coloured individuals of both species (W = 1.337, p = 0.247). On the other hand, activity pattern distributions showed significant differences between melanistic and non-melanistic clouded oncilla with a medium level of overlap (Δ = 0.74, 95% CI = 0.62 - 0.88, p = 0.04, Fig. 8B). The activity pattern distributions of melanistic clouded oncilla and margay did not differ significantly and showed a medium overlap (Δ = 0.74, 95% CI = 0.55 - 0.90, p = 0.152, Fig. 8C). The activity pattern distributions of non-melanistic clouded oncilla and margay did not differ significantly and showed high overlap (Δ = 0.87, 95% CI = 0.80 - 0.99, p = 0.929, Fig. 8D). Finally, the distributions of the complete data-set of clouded oncilla and margay did not differ significantly and showed high overlap (Δ = 0.82, 95% CI = 0.68 - 0.97, p = 0.791, Fig. 8A).

We include standout records of melanism in *Leopardus* species, with a significant proportion of daytime activity in camera trap records of melanistic individuals in comparison to non-melanistic ones. We also include proportions of all individual species records in comparison to unusual records per species (Fig. 4).

## Discussion

The increasing prevalence of unusual colouration in mammalian populations raises intriguing questions about the underlying causes and potential implications for wild species. Historically, records of albinism, melanism and other pigmentation disorders have been geographically localised, often tied to specific genetic pools or environmental conditions (Hubbard et al. 2010, Mooring et al. 2020). The records we reported comprise different unusual colourations. Some appear to be individual-specific, suggesting a low frequency of occurrence of mutations within the species population (e.g. Fig. 6D), whereas others have been recorded previously and appear to be more frequent in wild populations (e.g. Fig. 6E) (Ríos-Alvear and Cadena-Ortiz 2019, Cotts et al. 2023b). However, the proportion of independent records exhibiting melanistic individuals of the clouded oncilla allowed us to detect variations in activity levels, suggesting temporal segregation in crepuscular hours.

Camera traps are a low-invasive method for monitoring wildlife, allowing the detection of elusive species and types of behaviour that are difficult to observe using traditional sampling methods (Delisle et al. 2021). In this study, camera traps enabled us to identify unusual colouration phenotypes, which, alongside time data, allowed us to elucidate inter- and intraspecific activity pattern variations according to chromatic differences. However, image resolution, light and weather conditions influence the picture quality limiting a detailed identification of each colouration pattern (Lamoreux et al. 2010). We identified two types of chromatic disorders, hypopigmentation and hyperpigmentation, which refer to dark tones due to the high melanin concentration or lighter/pale tones resulting from low melanin concentration (Lamoreux et al. 2010, van Grouw 2006, van Grouw 2013, Lucati and López-Baucells 2017, van Grouw 2021, Cotts et al. 2023a, Cotts et al. 2023b). It is worth noting that genotype triggers phenotype, but in colour variation, various mutations lead to the same phenotype, even when the mutations affect different genes. For instance, the piebald gene is just one of the various genes affected by white spotting (van Grouw 2006, Lamoreux et al. 2010, van Grouw 2021). Thus, to identify a colour aberration, it is essential to characterise the underlying genetic base (Lamoreux et al. 2010).

The lack of pigmentation in wild mammals is generally considered a disadvantage, as it impacts the individual's camouflage, making it more visible to both predators and prey, therefore reducing its chances of successfully evading predators and hunting effectively (Caro 2005, van Grouw 2006, Abreu et al. 2013, Caro and Mallarino 2020, Aximoff et al. 2021, Streeting et al. 2023). However, we hypothesise that progressive greying in wild populations of the brown-nosed coati may either be relatively common at the individual level or result from a lack of genetic diversity within the group (van Grouw 2006, Lamoreux et al. 2010, Abreu et al. 2013). This is based on our observation of two brown-nosed coatis from the same group displaying this disorder, suggesting fitness implications (Guevara et al. 2011, Silva-Caballero et al. 2014) (Fig. 6B and C). White spotting/piebaldism is a locally disrupted melanocyte production, that could be tied to inheritable traits as well as environmental pressures (Lamoreux et al. 2010, Mello et al. 2020). We recorded three instances of white spotting/piebaldism in different species, the western mountain coati (Fig. 6D), the black agouti (Fig. 7A) and the tayra (Fig. 7B). While piebaldism has previously been documented in black agoutis in Ecuador (Mejía Valenzuela 2019, Fig. 1D), this is the first known record for the western mountain coati and the tayra, although the latter has been reported with leucistic individuals (Tortato and Althoff 2007, Talamoni et al. 2017). Information about the western mountain coati's colouration is limited; other authors have stated that the species' coat can vary from olive brown to grey, including pale tones with yellowish rings at the distal part of the tail (Helgen et al. 2009). To our knowledge, this is the first report of white spotting/piebaldism in western mountain coatis. However, leucism in the genus has been previously reported in *Nasuanarica*, although we suspect it is a xanthochromatic individual (Silva-Caballero et al. 2014). It is unkown the physiological implications of white spotting/piebaldism in western mountain coatis, particularly because genes affecting some unusual colouration patterns are pleitropic, meaning they intervene in other phenotypic processes related with the individual's normal development (Lamoreux et al. 2010). We consider that the whitish colouration observed in the southern tamandua is evidence of xanthochromism (Fig. 6D). The specimen has a pale colouration over most of its body, but the black face mask, ears and tail rule out problems regarding melanin production or transportation, which cause albinism and leucism, respectively (van Grouw 2006, Lamoreux et al. 2010, van Grouw 2013, Lucati and López-Baucells 2017, Cotts et al. 2023b). Despite previous records of colour variations in this species (Ríos-Alvear and Cadena-Ortiz 2019, Portillo et al. 2022, Cotts et al. 2023b), our report adds to the growing knowledge of its colouration patterns.

Coat colour polymorphism in felids has been extensively studied (Eizirik et al. 2003, Forsman et al. 2008, Schneider et al. 2015). The genetic basis of melanism in felids is well established, including its association with genes such as ASIP (Agouti Signalling Protein) and MC1R (Melanocortin-1 receptor), which regulate the expression of the melanistic phenotype (Kingsley et al. 2009), the former being recessive (i.e. a loss-of-function mutation) and the latter dominant (i.e. a gain-of-fuction mutation) (Schneider et al. 2015). However, it remains particularly intriguing to determine whether this mutation offers any advantage over non-melanistic individuals, while preserving intraspecific genetic diversity. Studies suggest that melanism has arisen independently multiple times within different felid lineages (Eizirik et al. 2003, Schneider et al. 2012, Schneider et al. 2015, Graipel et al. 2019), indicating that the evolutionary mechanisms behind it are complex and not fully understood (Schneider et al. 2015). Moreover, melanism appears to be as ancient as common colouration, closely linked to circadian habits (Graipel et al. 2019) and, due to its high prevalence in some wild felid species, it seemingly lacks deleterious effects (Schneider et al. 2015).

Graipel et al. (2019) recorded a total of 170 independent observations of oncillas (*Leopardustigrinus*) in Brazil, including 139 records with typical colouration and 31 melanistic records (i.e. 18%). In contrast, Mooring et al. (2020) reported 203 independent records of oncilla, including 65 melanistic records, representing 32% of the total observations. Our results include 127 independent records of the clouded oncilla, 44% of which are from melanistic individuals. These studies, conducted with similar sampling efforts, place our findings amongst the highest reported. It is worth noting that other pigmentary disorders may be confused with the melanistic phenotype. For example, pigment-type switching can result in the brown phenotype, which involves the overexpression of pheomelanin relative to eumelanin (Lamoreux et al. 2010). In this sense, camera-trap records limit our ability to make more detailed distinctions based solely on images. In addition, due to the recent taxonomic reorganisation of *Leopardus* spp. (Bonilla-Sánchez et al. 2024, de Oliveira et al. 2024), these data are relevant to *L.pardinoides*. The high proportion of melanistic *L.pardinoides* suggests that melanism may offer an advantage. Some authors have proposed that melanism in felids may be favoured by natural selection (Eizirik et al. 2003, Schneider et al. 2015), but the adaptive fitness in wild populations remains uncertain, warranting further investigation. One plausible explanation is that melanistic individuals may have access to more resources in distinct habitat niches, while not overlapping with other competitors (Forsman et al. 2008, Graipel et al. 2014).

Several hypotheses about melanism and its high frequency in wild felids are related to environmental variables such as humidity and temperature. It has been proposed that more humid environments improve thermoregulatory efficiency, as melanin pigments are better fixed, leading to a higher frequency of melanistic individuals in ecosystems with dense vegetation and high humidity (Eizirik et al. 2003, Mooring et al. 2020). Conversely, the temporal segregation hypothesis suggests that melanistic individuals may be more efficient at obtaining resources during bright nights compared to non-melanistic morphs, due to their greater cryptic colouration (Graipel et al. 2014, Mooring et al. 2020). Temporal segregation can occur within and between felid species (Graipel et al. 2014), which is consistent with our findings of a significant difference in activity patterns between melanistic and non-melanistic clouded oncillas.

Graipel et al. (2014) found that, although there was no clear trend towards nocturnal or diurnal activity in oncillas, they were more active on bright nights compared to other co-occurring felids such as margay and ocelots (*Leoparduspardalis*). In our study, activity levels and patterns between melanistic clouded oncilla and margay were similar, but there were differences in the activity patterns of melanistic versus non-melanistic clouded oncillas. Brighter nights may improve camouflage and access to resources for melanistic individuals compared to other syntopic competitors (Graipel et al. 2014). In contrast, we did not find differences in activity levels between melanistic and non-melanistic clouded oncillas, nor observed greater diurnal activity. However, we did observe higher diurnal activity compared to typically coloured clouded oncillas and margay (Fig. 8). Temporal segregation in melanistic clouded oncillas appears to be most significant during the three hours before sunrise, whereas non-melanistic clouded oncillas increase their activity around sunset. However, we did not explore the effects of disturbances on the activity patterns of wild felids since most of our records are from forested and protected areas, although other studies have suggested negative effects of human disturbances on wildlife activity patterns (Guerisoli et al. 2019).

The Llanganates-Sangay Connectivity Corridor hosts a rich diversity of ecosystems, spanning a wide elevation range (700–3812 m) (Ríos-Alvear et al. 2024). This provides favourable conditions for the co-occurrence of wildlife species from both the upper mountains and lowland regions. Additionally, its rugged topography fosters local endemism, with distinct species compositions even in areas that are geographically close (Jost 2004, Haynie et al. 2006, Reyes-Puig et al. 2022).

Our study contributes to the growing database on colouration patterns and their rates of occurrence in terrestrial mammals. However, it also raises intriguing questions about the mechanisms driving the prevalence of unusual colouration patterns and their effects on wild populations. For example, we observed similar proportions of melanistic and non-melanistic clouded oncillas on both sides of the corridor. This suggests that colour variations are widespread across the study area and may confer a survival advantage (Schneider et al. 2015). Given the key role of the CELS in promoting connectivity between wildlife populations, we propose that the observed variation in colouration patterns supports the corridor's effectiveness in facilitating genetic flow throughout the region. However, we recommend conducting a population genetic analysis to uncover the underlying causes of colour variations and their implications for the fitness of terrestrial mammals in the CELS.

## Figures and Tables

**Table 1.** Sources of information in our study.

Source	Year	N° of sampling stations	Sampling effort (trap/nights)	N° of records of unusual colouration patterns
Reyes-Puig and Ríos-Alvear (2013) and Ríos-Alvear and Reyes-Puig (2013)	2011–2012	10	1100	1
Reyes-Puig et al. (2023)	2019–2021	30	2532	1
This study (as part of Ríos-Alvear et al., unpublished data)	2022–2024	62	21475	53
This study: two casual encounters with melanistic *Leoparduspardinoides* (one dead and one run over)	2023–2024	-	-	2
